# CD8+ T-Cell Deficiency, Epstein-Barr Virus Infection, Vitamin D Deficiency, and Steps to Autoimmunity: A Unifying Hypothesis

**DOI:** 10.1155/2012/189096

**Published:** 2012-01-24

**Authors:** Michael P. Pender

**Affiliations:** ^1^School of Medicine, The University of Queensland, Brisbane, QLD 4072, Australia; ^2^Department of Neurology, Royal Brisbane and Women's Hospital, Brisbane, QLD 4029, Australia

## Abstract

CD8+ T-cell deficiency is a feature of many chronic autoimmune diseases, including multiple sclerosis, rheumatoid arthritis, systemic lupus erythematosus, Sjögren's syndrome, systemic sclerosis, dermatomyositis, primary biliary cirrhosis, primary sclerosing cholangitis, ulcerative colitis, Crohn's disease, psoriasis, vitiligo, bullous pemphigoid, alopecia areata, idiopathic dilated cardiomyopathy, type 1 diabetes mellitus, Graves' disease, Hashimoto's thyroiditis, myasthenia gravis, IgA nephropathy, membranous nephropathy, and pernicious anaemia. It also occurs in healthy blood relatives of patients with autoimmune diseases, suggesting it is genetically determined. Here it is proposed that this CD8+ T-cell deficiency underlies the development of chronic autoimmune diseases by impairing CD8+ T-cell control of Epstein-Barr virus (EBV) infection, with the result that EBV-infected autoreactive B cells accumulate in the target organ where they produce pathogenic autoantibodies and provide costimulatory survival signals to autoreactive T cells which would otherwise die in the target organ by activation-induced apoptosis. Autoimmunity is postulated to evolve in the following steps: (1) CD8+ T-cell deficiency, (2) primary EBV infection, (3) decreased CD8+ T-cell control of EBV, (4) increased EBV load and increased anti-EBV antibodies, (5) EBV infection in the target organ, (6) clonal expansion of EBV-infected autoreactive B cells in the target organ, (7) infiltration of autoreactive T cells into the target organ, and (8) development of ectopic lymphoid follicles in the target organ. It is also proposed that deprivation of sunlight and vitamin D at higher latitudes facilitates the development of autoimmune diseases by aggravating the CD8+ T-cell deficiency and thereby further impairing control of EBV. The hypothesis makes predictions which can be tested, including the prevention and successful treatment of chronic autoimmune diseases by controlling EBV infection.

## 1. Introduction

**Figure 1:**
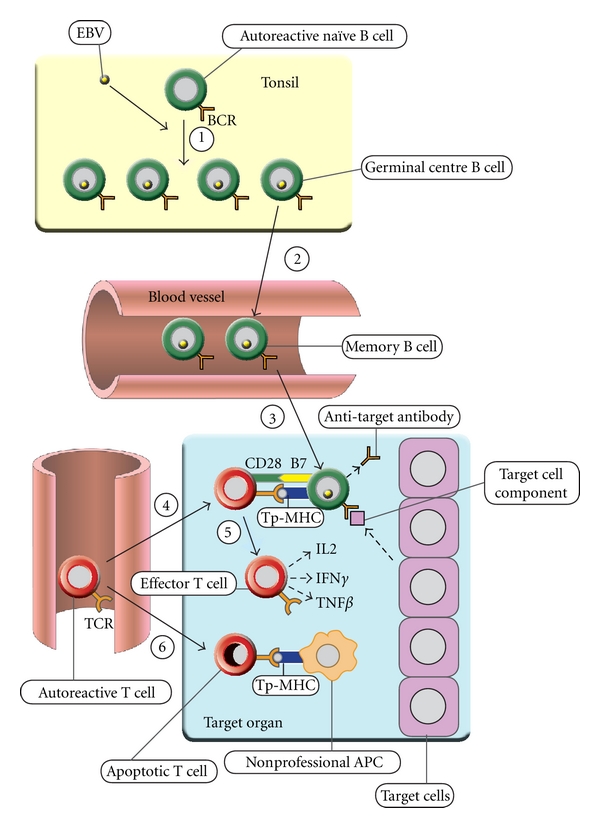
Proposed role of EBV infection in the development of chronic autoimmune diseases. During primary infection EBV infects autoreactive naïve B cells in the tonsil, driving them to enter germinal centres where they proliferate and differentiate into latently infected autoreactive memory B cells (path 1) which then exit from the tonsil and circulate in the blood (path 2). The number of EBV-infected B cells is normally controlled by EBV-specific cytotoxic CD8+ T cells, which kill proliferating and lytically infected B cells, but not if there is a defect in this defence mechanism. Surviving EBV-infected autoreactive memory B cells enter the target organ where they take up residence and produce oligoclonal IgG and pathogenic autoantibodies which attack components of the target organ (path 3). Autoreactive T cells that have been activated in peripheral lymphoid organs by cross-reacting foreign antigens circulate in the blood and enter the target organ where they are reactivated by EBV-infected autoreactive B cells presenting target organ peptides (Tp) bound to major histocompatibility complex (MHC) molecules (path 4). These EBV-infected B cells provide costimulatory survival signals (B7) to the CD28 receptor on the autoreactive T cells and thereby inhibit the activation-induced T-cell apoptosis which normally occurs when autoreactive T cells enter the target organ and interact with nonprofessional antigen-presenting cells (APCs) which do not express B7 costimulatory molecules [168, 169] (Path 6). After the autoreactive T cells have been reactivated by EBV-infected autoreactive B cells, they produce cytokines such as interleukin-2 (IL2), interferon-*γ* (IFN*γ*) and tumour necrosis factor-*β* (TNF*β*) and orchestrate an autoimmune attack on the target organ (Path 5). BCR, B cell receptor; TCR, T cell receptor.

**Figure 2:**
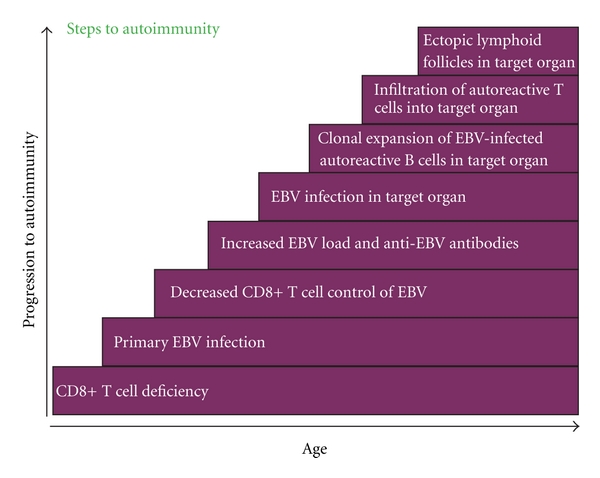
Steps to autoimmunity.

Epstein-Barr virus (EBV) has been suspected of involvement in the pathogenesis of various chronic autoimmune diseases since the finding of elevated levels of antibody to the virus in systemic lupus erythematosus (SLE) in 1971 [1]. Generally the effect of EBV infection has been attributed to immunological cross-reactivity between EBV and self-antigens [2–4]; however, in 2003 the EBV-infected autoreactive B-cell hypothesis of autoimmunity was proposed as the basis for human chronic autoimmune diseases [5]. This hypothesis proposes that, in genetically susceptible individuals, EBV-infected autoreactive B cells seed the target organ where they produce pathogenic autoantibodies and provide costimulatory survival signals to autoreactive T cells which would otherwise die in the target organ by activation-induced apoptosis [5] (Figure 1). The present article presents a further development of this hypothesis, proposing that susceptibility to develop chronic autoimmune diseases after EBV infection is dependent on a genetically determined quantitative deficiency of the cytotoxic CD8+ T cells that normally keep EBV infection under tight control. It is postulated that autoimmunity evolves in the following steps: (1) CD8+ T-cell deficiency; (2) primary EBV infection; (3) decreased CD8+ T-cell control of EBV; (4) increased EBV load and increased anti-EBV antibodies; (5) EBV infection in the target organ; (6) clonal expansion of EBV-infected autoreactive B cells in the target organ; (7) infiltration of autoreactive T cells into the target organ; and (8) development of ectopic lymphoid follicles in the target organ (Figure 2).

The evidence for each of these steps is considered in turn after a general introduction to EBV. There follows a summary of the proposed scenario for the development of autoimmune diseases and suggested strategies for testing the hypothesis.

## 2. EBV

EBV is a ubiquitous human herpesvirus that has the unique ability to infect, activate, and latently persist in B lymphocytes for the lifetime of the infected individual. During primary infection, EBV transmitted via saliva infects naïve B cells in the tonsil through the binding of the viral surface glycoprotein gp350 to complement receptor 2 (also known as CD21), which is expressed by mature B-cells and follicular dendritic cells [6]. EBV drives the infected B cell out of the resting state to become an activated B blast and then exploits the normal pathways of B cell differentiation so that the B blast differentiates in a germinal centre to become a latently infected resting memory B cell which exits from the germinal centre and circulates in the blood [7]. Latently infected memory B cells returning to the tonsil can terminally differentiate into plasma cells, which initiates the lytic (replicative) cycle with the production of infectious virus [8]. The resulting free virions infect tonsil epithelial cells where the virus replicates at a high rate and is continuously shed into saliva for transmission to new hosts [9]. Newly formed virus can also infect additional naïve B cells in the same host.

Latently infected memory B cells display the molecular hallmarks of classical antigen-selected memory B cells, namely, somatic hypermutation and class-switch recombination of their immunoglobulin (Ig) genes [10]. In normal B-cell differentiation, naïve B cells are activated by antigen through the B-cell receptor (BCR) and by T-cell help through the CD40 receptor so that they proliferate and progress through a germinal centre reaction. Remarkably, EBV expresses two proteins, latent membrane protein 2A (LMP2A) and LMP1, which mimic the antigen-activated BCR and the activated CD40 receptor, respectively [11, 12]. In the tonsil LMP2A and LMP1 appear to work synergistically with BCR signalling and CD40 signalling, respectively [13].

EBV infection is normally kept under tight control by EBV-specific immune responses, especially by cytotoxic CD8+ T cells which eliminate proliferating and lytically infected B cells [14]. In the developing world most children become infected within the first three years of life, and EBV seropositivity reaches 100% within the first decade [15]. These early primary infections are almost always asymptomatic. In contrast, in the developed world, up to half the children are still EBV seronegative at the end of their first decade and subsequently become infected through intimate oral contact in adolescence or young adulthood [15]. As many as 50% of these delayed primary infections are symptomatic and manifest as acute infectious mononucleosis. 

## 3. Steps to Autoimmunity

### 3.1. Step 1: CD8+ T-Cell Deficiency

Since 1980 it has been recognized that the proportion and number of CD8+ T cells in the peripheral blood are decreased and that the CD4/CD8 ratio is increased in patients with autoimmune diseases, including multiple sclerosis (MS) [16–21], rheumatoid arthritis (RA) [22, 23], SLE [24, 25], Sjögren's syndrome [25, 26], systemic sclerosis [27, 28], dermatomyositis [29, 30], primary biliary cirrhosis [31], primary sclerosing cholangitis [32], ulcerative colitis [33, 34], Crohn's disease [33], psoriasis [35], vitiligo [36, 37], bullous pemphigoid [38, 39], alopecia areata [40], idiopathic dilated cardiomyopathy [41, 42], type 1 diabetes mellitus [43–46], Graves' disease [47, 48], Hashimoto's thyroiditis [47, 49], myasthenia gravis [50, 51], IgA nephropathy [52, 53], membranous nephropathy (or membranous glomerulonephritis) [52, 53], and pernicious anaemia [54, 55]. Although some studies have not found CD8+ T-cell deficiency in patients with autoimmune diseases [56] or have attributed the deficiency to hormonal factors [57], CD8+ T-cell deficiency would appear to be a general feature of human chronic autoimmune diseases. This was initially interpreted as a decrease in suppressor CD8+ T cells leading to disinhibition of autoimmune responses [16, 22, 24, 47, 50] but later attributed to sequestration of CD8+ T cells in the target organ [19, 23, 31] because CD8+ T cells are selectively enriched compared to CD4+ T cells in the target organ in some autoimmune diseases [23, 58]. However, if CD8+ T cells are accumulating in the target organ because of the presence of EBV, the number of CD8+ T cells in the blood should increase, not decrease, because normally the CD8+ T-cell response increases with EBV load [59–61]. An alternative explanation, and the one proposed here, is that a genetic deficiency of CD8+ T cells results in a decreased CD8+ T-cell response to EBV, which allows EBV-infected B cells to accumulate in the target organ.

**Figure 3:**
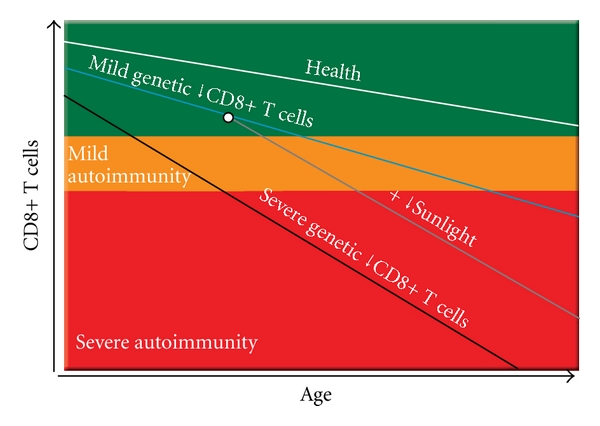
Proposed genetic deficiency of CD8+ T cells underlying the development of chronic autoimmune diseases. The upper green panel on the graph represents health, the middle orange panel, the development of mild autoimmune disease (mild autoimmunity) and the lower red panel the development of severe progressive autoimmune disease (Severe autoimmunity). In normal individuals (Health) the number of CD8+ T cells declines with increasing age but still remains sufficient to control EBV infection. In individuals with a mild genetic deficiency of CD8+ T cells, the deficiency is aggravated by increasing age eventually leading to insufficient CD8+ T cells to control EBV infection. In individuals carrying HLA class II or class I genes predisposing to specific autoimmune diseases, this leads to the accumulation of EBV-infected B cells in the target organ and the development of autoimmune disease, which progresses in severity as the CD8+ T-cell count further declines with age and as the EBV load in the target organ subsequently increases. In individuals with a severe genetic deficiency of CD8+ T cells, autoimmune diseases develop at a younger age and progress more rapidly. Deprivation of sunlight and vitamin D at higher latitudes aggravates the genetic CD8+ T-cell deficiency and increases the incidence and progression of autoimmune disease.

The CD4/CD8 T-cell ratio in humans is genetically controlled [62], with at least some of the responsible genes being located in the HLA complex [63]. The CD8+ T cell deficiency and increased CD4/CD8 ratio in autoimmune diseases are also present in the healthy blood relatives of patients with these diseases [36, 45, 46, 64, 65], indicating that the abnormalities are genetically determined and not secondary to the disease process. Interestingly, females generally have lower proportions and numbers of CD8+ T cells, higher proportions and numbers of CD4+ T cells, and higher CD4/CD8 ratios than males [62, 66–70]. These gender differences appear to be hormonally mediated because oestrogen deficiency substantially increases the proportion and number of CD8+ T cells and decreases the CD4/CD8 ratio, with the ratio directly correlating with the serum oestradiol level [71]. Lower numbers of CD8+ T cells in females might contribute to the higher frequency of autoimmune diseases in females than males. Because the number of CD8+ T cells normally declines with increasing age, particularly through childhood [72], but also through adulthood [62, 70, 73], the primary CD8+ T cell deficiency will be aggravated as each person ages, as occurs in patients with MS [74] (Figure 3).

Exposure to natural sunlight or treatment in a solarium increases the proportion of CD8+ T cells and decreases the CD4/CD8 T cell ratio in peripheral blood [75–77]. Exactly how sunlight increases the number of CD8+ T cells is unclear, but the effect is probably mediated at least in part by vitamin D because (1) among cells of the immune system, activated CD8+ T cells express the highest concentrations of the vitamin D receptor [78]; (2) vitamin D increases the mitogen-induced proliferation of CD8+ T cells and decreases the CD4/CD8 ratio in bovine peripheral blood mononuclear cells in vitro [79]; (3) vitamin D administration increases the CD8+ T cell count [80]; (4) vitamin D deficiency is associated with a decreased proportion of CD8+ T cells and increased CD4/CD8 ratio [81]. Here it is proposed that deprivation of sunlight and vitamin D aggravates the genetically determined CD8+ T cell deficiency and impaired control of EBV infection and thereby contributes to the high prevalence of autoimmune diseases such as MS, type 1 diabetes, and RA at high latitudes [82–84] (Figure 3) and that the protective effect of vitamin D against autoimmune diseases [85–87] is mediated at least in part by an increase in the number of CD8+ T cells available to control EBV. A higher frequency of late primary EBV infection at higher latitude might also contribute to the latitudinal gradient [88].

### 3.2. Step 2: Primary EBV Infection

Patients with MS are almost universally (>99%) seropositive for EBV, but not for other viruses [89, 90]. In a meta-analysis of 13 case-control studies comparing EBV serology in MS patients and controls, 99.5% of MS patients were EBV seropositive compared to 94.0% of controls, with EBV seronegativity having an OR_MH_ odds ratio of MS of 0.06 (exact 95% CI: 0.03, 0.13; *P* < 0.000000001) [91]. Serial studies have shown that the risk of developing MS is extremely low among individuals not infected with EBV but increases sharply in the same individuals following EBV infection, with an estimated mean interval of 5.6 years between primary EBV infection and onset of MS [92]. These studies suggest that EBV infection is essential for the development of MS but, by itself, is not sufficient to cause MS because the vast majority of people infected with EBV do not develop the disease [93]. The dramatic increase in MS risk following primary EBV infection [92] is obscured by the fact that EBV infects such a large proportion (~95%) of the general adult population. If EBV does have an essential role in the development of MS, studies in children should show a larger numerical difference in the frequency of EBV seropositivity between MS patients and controls because the prevalence of EBV infection in the general population is considerably lower in children than in adults. Indeed, children with MS have an EBV-seropositivity rate of 86–99% compared to 64–72% in age-matched controls [94–96].

As with MS, virtually all patients with SLE are seropositive for EBV [97, 98]. This is particularly striking in children and young adults where 99% of patients with SLE are EBV seropositive compared to 70% of age-matched controls (*P* < 0.00000000001) [97]. The above findings strongly suggest that EBV infection is a prerequisite for the development of MS and SLE, raising the possibility that the same might apply to other chronic autoimmune diseases [5]. Recent studies have found all children with autoimmune hypothyroidism to be EBV seropositive compared to 51.6% of age-matched controls [99] and 98.5% of adults with Graves' disease to have antibodies to EBV nuclear antigens compared to 78.1% of controls [100]. All patients with coeliac disease were also found to be EBV seropositive in one study although the seropositivity rate in controls was not documented [101]. Studies of EBV seroprevalence are lacking in other autoimmune diseases.

The age at which primary infection with EBV occurs is also important. As discussed above in Section 2, when primary EBV infection occurs in early childhood, as it generally does in the developing world, it is asymptomatic but when it is delayed to the time of adolescence or later, as in the developed world, it commonly manifests as infectious mononucleosis where the number of latently infected memory B cells can rise to half, and perhaps even higher, of the peripheral memory B cell compartment [102]. Why a higher proportion of B cells should be infected when primary infection is delayed beyond childhood to adolescence or later is unclear. Possible explanations include a higher dose of viral inoculum acquired by intimate oral contact and a reduced capacity to mount a rapid effective CD8+ T-cell response in adolescents/adults compared to young children. The absolute size of the CD8+ T-cell population in healthy individuals decreases threefold between the ages of 2 and 16 years [72]. Here it is proposed that a genetically determined CD8+ T-cell deficiency does not lead to impaired control of EBV when primary infection occurs in early childhood, unless the deficiency is severe, but that when primary infection occurs in adolescence or adulthood after the normal age-related precipitous decline in CD8+ T cells the same genetic CD8+ T-cell deficiency is more likely to impair control of EBV infection. This might explain why infectious mononucleosis increases the risk of MS [103] and why the prevalence of MS is high when primary infection with EBV is delayed beyond puberty [104]. The occurrence of EBV infection at a younger age in children from less prosperous socioeconomic groups [105] is likely to be an important contributor to the protection that substandard hygiene confers against autoimmunity [106].

### 3.3. Step 3: Decreased CD8+ T-Cell Control of EBV

EBV infection is normally kept under tight control by EBV-specific immune responses, especially by cytotoxic CD8+ T cells, which eliminate proliferating and lytically infected B cells [14]. CD8+ T cell control of EBV-infected B cells is impaired in patients with autoimmune diseases, including RA [107–109], SLE [110], Sjögren's syndrome [111, 112], MS [113, 114], primary biliary cirrhosis [115], and systemic sclerosis [116]. In MS this defective control of EBV is not due to increased B cell resistance to killing by cytotoxic CD8+ T cells or to a functional impairment in the cytotoxic ability of CD8+ T cells but results from a decrease in the number of EBV-specific CD8+ T cells [114]. This in turn stems from the general deficiency of CD8+ T cells and from a decreased proportion of EBV-specific T cells within the total CD8+ T-cell population [74]. The low proportion of EBV-specific T cells within the CD8+ T-cell population in MS has been proposed to be due to T-cell exhaustion [74], which occurs in virus-specific CD8+ T cells during high-grade chronic viral infections [117]. Deprivation of sunlight and vitamin D will also aggravate the CD8+ T-cell deficiency and impaired control of EBV, as discussed above in Section 3.1.

### 3.4. Step 4: Increased EBV Load and Increased Anti-EBV Antibodies

The EBV DNA load, as measured by the total number of viral genomes, is increased in the blood of patients with autoimmune diseases, including Sjögren's syndrome [118, 119], RA [120], SLE [121], primary biliary cirrhosis [122], and inflammatory bowel disease [123]. Patients with RA [109] and SLE [124] also have been shown to have an increased frequency of EBV-infected B cells in their blood, but whether this is the case in other autoimmune diseases has not been determined.

The level of anti-EBV antibodies in the blood is also increased in autoimmune diseases, including SLE [1], RA [125], MS [126], idiopathic pulmonary fibrosis (or cryptogenic fibrosing alveolitis) [127], Sjögren's syndrome [128], IgA nephropathy [129] and autoimmune thyroid disease [130]. Elevation of anti-EBV antibodies precedes the onset and increases the risk of MS [131–133]. Here it is proposed that the increase in anti-EBV antibodies reflects an increased EBV load consequent to the decreased CD8+ T-cell response to EBV. The production of anti-EBV antibodies may also be enhanced by increased help from CD4+ T cells, which show increased reactivity to EBV in SLE [121] and MS [134].

### 3.5. Step 5: EBV Infection in the Target Organ

In healthy EBV-seropositive subjects, the frequency of EBV-infected B cells in the peripheral blood is ~5 per 10^6^ B cells [135]. Thus it may be anticipated that a similar low frequency of EBV-infected B cells will be found at any site of tissue inflammation involving B cells, regardless of cause. For EBV-infected B cells in the target organ to be incriminated in the pathogenesis of autoimmunity, they should be found at a substantially higher frequency than would occur if the proportion of EBV-infected B cells within the B-cell component of the tissue infiltrate simply reflected that in the blood. The gold standard for detection of EBV-infected B cells in histological material is in situ hybridization for EBV-encoded small RNA (EBER-ISH) [136], which allows determination of the frequency of EBV-infected B cells in B-cell infiltrates. However, if the tissue viral load is defined as the total EBV genome copy number determined by the polymerase chain reaction (PCR), then it is not possible to determine whether a large increase in viral load is due to a large increase in the frequency of latently infected B cells, which express only two to five copies of the viral genome per cell, or a very small increase in the fraction of infected cells replicating the virus, which contain thousands of genomes per cell [124]. The probability of detecting EBV in the target organ is also influenced by the size of the tissue sample, with a lower probability of detection in biopsies than in studies of the whole organ [137].

Studies using EBER-ISH have found an increased frequency of EBV-infected B cells in the salivary glands in Sjögren's syndrome [138, 139], the colon in ulcerative colitis and Crohn's disease [137, 140], the brain in MS [141], the thymus in myasthenia gravis [142], and the thyroid gland in Graves' disease [100]. Studies using PCR alone have shown increased levels of EBV DNA in the liver in primary biliary cirrhosis [122], kidney in IgA nephropathy and membranous nephropathy [143], and lung in idiopathic pulmonary fibrosis [144].

Whereas the concept of a target organ is clear in organ-specific autoimmunity, it is less so in systemic autoimmunity. In SLE, for example, tissue damage can be mediated either by autoantibodies produced in lymphoid organs remote from the targeted tissue or by direct lymphocytic infiltration of nonlymphoid organs such as the kidney [145]. Thus for Steps 5, 6, and 8 in systemic autoimmunity the term “target organ” is extended to encompass not only targeted nonlymphoid tissues infiltrated with lymphocytes, such as the kidney, but also *targeting* lymphoid organs such as the bone marrow, lymph nodes, and spleen which may be sites of clonal expansion of EBV-infected autoreactive B cells.

### 3.6. Step 6: Clonal Expansion of EBV-Infected Autoreactive B Cells in the Target Organ

Monoclonal or oligoclonal B-cell expansion occurs in the thyroid gland in Hashimoto's thyroiditis [146], salivary glands in Sjögren's syndrome [147, 148], synovium in RA [149, 150], cerebrospinal fluid in MS [151], liver in primary biliary cirrhosis [152], muscle in dermatomyositis and polymyositis [153], and blood in SLE [154]. These clonally expanded B cells exhibit the molecular hallmarks of an antigen-driven germinal centre reaction, namely, somatic hypermutation and high replacement-to-silent mutation ratios in the complementarity-determining regions of the Ig variable (V) region genes [148, 150, 151]. Autoreactive B cells have been demonstrated in the thyroid gland in autoimmune thyroid disease [155, 156], salivary glands in Sjögren's syndrome [157], and liver in primary biliary cirrhosis [158].

B-cell clonal expansion within the target organ has been proposed to be due to EBV infection of autoreactive B cells [5]. The probability of EBV infecting autoreactive B cells is not low because at least 20% of human naïve B cells are autoreactive [159]. Indeed, EBV infection of naïve B cells of normal individuals in vitro results in the production of monoclonal autoantibodies [160]. In healthy individuals, most of the autoantibodies produced by autoreactive B cells in vivo are of the IgM class and are nonpathogenic or “natural” antibodies [161]. However, in individuals with CD8+ T cell deficiency, uncontrolled infection of naïve autoreactive B cells by EBV in vivo could drive these cells through a germinal centre reaction, with somatic hypermutation and class-switch recombination resulting in the production of pathogenic IgG or IgA autoantibodies. Memory B cells latently infected with EBV display the same molecular hallmarks of an antigen-driven germinal centre reaction [10] as those exhibited by clonally expanded B cells isolated from target organs. As yet it has not been determined whether clonally expanded and autoreactive B cells in target organs are infected with EBV.

### 3.7. Step 7: Infiltration of Autoreactive T Cells into the Target Organ

Infiltration of autoreactive T cells into the target organ has been demonstrated by the isolation of thyroid-specific T cells from the thyroid gland in Graves' disease [162, 163], pyruvate dehydrogenase complex-specific T cells from the liver in primary biliary cirrhosis [164, 165], Ro(SSA)-specific T cells from the salivary glands in Sjögren's syndrome [166], and T cells specific for transglutaminase-modified gliadin from duodenal mucosa in coeliac disease [167]. In the case of primary biliary cirrhosis it has been shown that there is a marked enrichment of autoreactive CD4+ T cells and CD8+ T cells in the liver compared to the peripheral blood [164, 165]. Here it is proposed that, after activation in peripheral lymphoid organs by cross-reacting foreign antigens, autoreactive T cells enter the target organ where they are reactivated by EBV-infected B cells which present self-antigens and provide costimulatory survival signals, thereby inhibiting the activation-induced T-cell apoptosis which normally occurs when autoreactive T cells enter the target organ [168, 169] (Figure 1). It is postulated that the infiltrating autoreactive T cells then orchestrate an immune attack on the target organ through the recruitment of macrophages and additional B cells.

### 3.8. Step 8: Development of Ectopic Lymphoid Follicles in the Target Organ

A frequent finding in organs targeted by autoimmunity is the presence of ectopic lymphoid tissue containing B-cell follicles with germinal centres, which may represent sites of clonal expansion of autoreactive B cells specific for antigens present in the target organ. Such ectopic B-cell follicles are found in the gut in Crohn's disease [170] and ulcerative colitis [171], the liver in primary biliary cirrhosis [172], the thyroid gland in Hashimoto's thyroiditis [156, 173] and Graves' disease [156], the synovium in RA [174, 175] and psoriatic arthritis [176], the lung in idiopathic pulmonary fibrosis [177, 178], the salivary glands in Sjögren's syndrome [157, 179], the brain in MS [180, 181], the kidney in membranous nephropathy [182] and IgA nephropathy [183], and muscle in dermatomyositis [184]. Autoreactive B cells have been identified within these ectopic lymphoid follicles by their ability to bind biotinylated self-antigens: thyroid antigens in the thyroid gland in Hashimoto's thyroiditis and Graves' disease [156] and Ro and La nuclear antigens in the salivary glands in Sjögren's syndrome [157].

In myasthenia gravis, where weakness is usually mediated by antibodies to the acetylcholine receptor, there is minimal lymphocytic infiltration at the neuromuscular junction, but the thymus resembles an autoimmune target organ because it contains ectopic B-cell follicles with germinal centres [185, 186] lying adjacent to thymic myoid cells expressing the acetylcholine receptor [187]. In SLE, which is characterized by the production of non-organ-specific autoantibodies, follicular hyperplasia occurs in the lymph nodes [188]. These lymph node hyperplastic germinal centres may represent sites of clonal expansion of autoreactive B cells reactive to ubiquitous self-antigens, such as those present in the nuclei of all cells.

Here it is proposed that ectopic lymphoid follicles are major sites of EBV persistence in chronic autoimmune diseases, as has been shown in Sjögren's syndrome [138], inflammatory bowel disease [140], MS [141], and myasthenia gravis [142].

## 4. Proposed Scenario for the Development of Chronic Autoimmune Diseases

**Figure 4:**
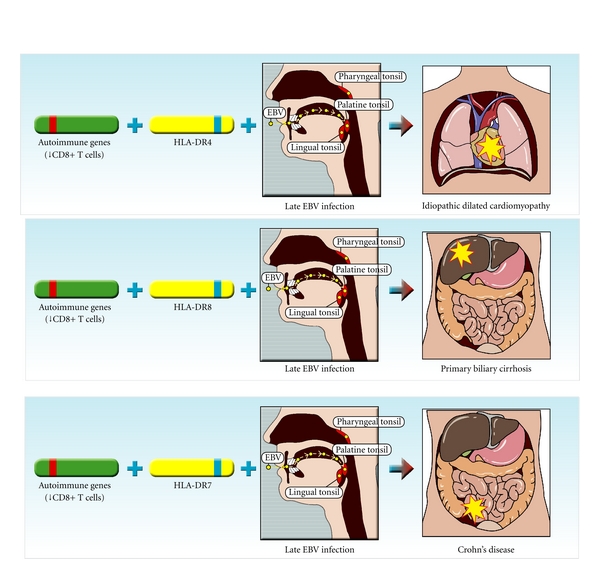
Proposed sequence of events leading to the development of chronic autoimmune diseases. In individuals with a genetic deficiency of CD8+ T cells (carried by “Autoimmune genes”) and with HLA class II genes predisposing to idiopathic dilated cardiomyopathy (HLA-DR4 [206]), primary biliary cirrhosis (HLA-DR8 [207]), and Crohn's disease (HLA-DR7 [208]), primary EBV infection, particularly if delayed (Late), leads to the infection of autoreactive B cells, which accumulate in the target organ where they reactivate autoreactive T cells that orchestrate an autoimmune attack on the organ. For simplicity these depictions focus on the role of CD8+ T-cell deficiency, EBV infection and selected HLA alleles and do not include interactions with other genetic and environmental factors that may also contribute to the pathogenesis of autoimmune diseases.

The genetic background of autoimmunity is clearly complex and involves epistatic interactions between genes [189] and epigenetic modification of gene expression [190]. Genetic factors contributing to the development of chronic autoimmune diseases can be divided into those that confer a general predisposition to autoimmunity and those that confer susceptibility to specific autoimmune diseases. A general predisposition to autoimmunity is manifested by the increased occurrence of various autoimmune diseases in individuals with a given autoimmune disease and in their blood relatives [191–195]. Indeed there is evidence that this general predisposition to autoimmunity is inherited as a Mendelian dominant trait [191]. Here it is proposed that genetically determined CD8+ T-cell deficiency, with consequent impairment of CD8+ T-cell control of EBV-infected B cells, is the mechanism underlying this general predisposition to autoimmunity (Figures 3 and 4). For genes conferring susceptibility to specific autoimmune diseases, the most widely characterized are specific alleles of HLA class II and less frequently HLA class I genes [196]. The mechanism underlying this is unclear but the most likely explanation is that the HLA molecules encoded by the specific alleles determine which self-antigens (and therefore which organs) are recognized by T cells that have been activated by crossreacting foreign antigens or modified self antigens. The following scenario describes how a genetic deficiency of CD8+ T cells might lead to the development of chronic autoimmune diseases after infection with EBV. This is based on the EBV-infected autoreactive B cell hypothesis of autoimmunity, which proposes that, in genetically susceptible individuals, EBV-infected autoreactive B cells seed the target organ where they produce pathogenic autoantibodies and provide costimulatory survival signals to autoreactive T cells [5]. It is important to clarify here that what is proposed is that EBV is essential (through infection of autoreactive B cells), but not necessarily the only environmental agent required for the development of autoimmune diseases. For example, gluten exposure is a prerequisite for coeliac disease. Particular infectious agents other than EBV might also be needed to activate autoreactive T cells through molecular mimicry in particular autoimmune diseases.

A genetic deficiency of CD8+ T cells would exert its effect from the time of primary EBV infection in the tonsil by prolonging the survival and proliferation of infected B lymphoblasts and germinal centre B cells and the survival of plasma cells replicating the virus. This would increase the probability of clonal expansion of EBV-infected autoreactive B cells. It is proposed that EBV-infected autoreactive B cells lodge and persist in the organ containing the self antigen they recognize. Depending on the disease-specific HLA class II or class I alleles carried by a particular person, exposure to foreign agents (including EBV itself) leads to the activation of T cells which cross-react with self antigens and traffic into the organ containing these self antigens where they are reactivated by EBV-infected B cells presenting self antigens (Figures 1 and 4). These EBV-infected B cells produce pathogenic autoantibodies and also provide costimulatory survival signals to the autoreactive T cells, thereby inhibiting the activation-induced T-cell apoptosis which normally occurs when autoreactive T cells enter the target organ [168, 169]. The autoreactive T cells orchestrate an immune attack on the target organ through the recruitment of macrophages and additional B cells. Self antigens released by this attack lead to spreading of autoreactivity to other autoantigens in the target organ. Repeated T-cell attacks on the target organ supported by local EBV-infected B cells lead to the development, within the target organ, of ectopic B-cell follicles with germinal centres generating more autoreactive B cells. The autoimmune process itself could foster the survival and proliferation of EBV-infected autoreactive B cells in the target organ by releasing self antigens and giving CD4+ T-cell-help, which would complement the BCR and CD40 receptor signalling already provided by EBV-encoded LMP2A and LMP1, respectively [197]. This could lead to a vicious circle wherein EBV-infected autoreactive B cells promote autoimmunity, which in turn promotes EBV infection in the target organ. With prolonged high EBV load, T-cell exhaustion would supervene, further compromising the CD8+ T-cell control of EBV and further increasing the EBV load. Deprivation of sunlight and vitamin D would also aggravate the CD8+ T-cell deficiency and impaired control of EBV (Figure 3).

## 5. Testing the Hypothesis

### 5.1. Is EBV Infection Necessary for the Development of Chronic Autoimmune Diseases?

If EBV infection is necessary for the development of chronic autoimmune diseases it should be possible to prevent and successfully treat these diseases by controlling EBV infection [198].

#### 5.1.1. Prevention

Vaccination of healthy EBV-seronegative young adults with recombinant gp350 is effective in preventing the development of infectious mononucleosis induced by EBV infection, although it does not prevent asymptomatic EBV infection [199]. The vaccinated subjects showed seroconversion to anti-gp350 antibodies which persisted >18 months and were probably responsible for the protective effect because anti-gp350 antibody neutralizes EBV infectivity [200]. Vaccination with gp350 might decrease the incidence of chronic autoimmune diseases by reducing the number of B cells infected by EBV, and thereby decreasing the probability of infected autoreactive B cells, during primary infection.

#### 5.1.2. Treatment

There are potentially 3 ways to treat chronic autoimmune diseases by controlling EBV infection: (1) B-cell depletion with monoclonal antibodies; (2) boosting immunity to EBV; (3) antiviral drugs. B-cell depletion with rituximab eliminates not only EBV-infected B cells but also uninfected B cells, which normally confer protective immunity against infectious agents. Improvement of an autoimmune disease with rituximab therapy would be consistent with an essential role of EBV in the development of the disease but would not constitute proof because the beneficial effect could be mediated by the elimination of autoreactive B cells not infected with EBV. More convincing evidence for an essential role of EBV would be eradication of autoimmune diseases by boosting immunity to EBV or by treatment with antiviral drugs. Humoral immunity to EBV could be boosted by vaccination with gp350 or administration of humanized or human monoclonal antibody against gp350. CD8+ T-cell immunity could be boosted by the intravenous infusion of autologous EBV-specific cytotoxic CD8+ T cells after expansion in vitro [201] or by the administration of agents such as interleukin-7, which expands the population of functional virus-specific CD8+ T cells in chronic viral infection [202]. With regard to antiviral drugs, treatment with aciclovir and related drugs, which inhibit herpesvirus DNA polymerase, is likely to have only a limited beneficial effect in chronic autoimmune diseases because these drugs act on EBV only when it is using its own DNA polymerase to replicate its DNA. This will apply only to lytically infected cells but not to latently infected ones, which replicate EBV DNA through the use of EBV nuclear antigen 1 (EBNA1) to engage host cell DNA polymerase. One strategy to overcome this would be first to administer rituximab to eliminate as many EBV-infected B cells as possible and to follow this with long-term antiviral drug therapy. An alternative approach is to target LMP1 [203], LMP2A, or EBNA1 [204] to inhibit EBV in latently infected cells. It has also been suggested that retroviral integrase inhibitors might be effective against EBV in autoimmune diseases [205]. If EBV infection of B cells in the target organ underpins the development of autoimmune diseases, effective antiviral drugs have the potential to be curative.

### 5.2. Are EBV-Infected B Cells in the Target Organ Autoreactive?

Whether EBV-infected B cells in the target organ are autoreactive could be addressed by determining whether they bind biotinylated self antigens in the same way that intrathyroidal germinal centre B cells specifically bind thyroid antigens in autoimmune thyroid disease [156].

### 5.3. Does CD8+ T-Cell Deficiency Underlie the Development of Chronic Autoimmune Diseases following EBV Infection?

Whether CD8+ T cell deficiency underlies the development of chronic autoimmune diseases following EBV infection could be addressed by the following experiments: (1) prospective studies to determine whether CD8+ T cell deficiency precedes the development of autoimmune diseases; (2) determining whether genetic variants associated with a decreased number of CD8+ T cells, such as the A allele of rs2524054 in HLA-B [63], predispose to chronic autoimmune diseases; (3) examining whether autoimmune diseases can be successfully treated by the intravenous infusion of autologous EBV-specific cytotoxic CD8+ T cells after expansion in vitro or by the administration of interleukin-7 to boost CD8+ T-cell immunity.

### 5.4. Does Vitamin D Deficiency Contribute to the Development of Autoimmune Diseases by Depleting CD8+ T Cells?

Whether vitamin D deficiency contributes to the development of autoimmune diseases by depleting CD8+ T cells could be tested by determining whether treatment of vitamin D deficiency in patients by dietary supplementation or exposure to sunlight increases the CD8+ T cell response to EBV, decreases the EBV load and produces clinical improvement.

## 6. Conclusions

CD8+ T-cell deficiency is a general feature of chronic autoimmune diseases and also occurs in healthy blood relatives of patients with these diseases. It is proposed that this deficiency is genetically determined and underlies the development of chronic autoimmune diseases by impairing CD8+ T-cell control of EBV infection, with the result that EBV-infected autoreactive B cells accumulate in the target organ where they produce pathogenic autoantibodies and provide costimulatory survival signals to autoreactive T cells. Autoimmunity is postulated to evolve in a series of steps culminating in the development of ectopic lymphoid follicles containing EBV-infected autoreactive B cells in the target organ. It is also proposed that deprivation of sunlight and vitamin D facilitates the development of autoimmune diseases by aggravating the CD8+ T cell deficiency and thereby further impairing control of EBV. The hypothesis makes predictions which can be tested, including the prevention and successful treatment of chronic autoimmune diseases by controlling EBV infection.

## References

[1] Evans AS, Rothfield NF, Niederman JC (1971). Raised antibody titres to E.B. virus in systemic lupus erythematosus. *The Lancet*.

[2] Lang HLE, Jacobsen H, Ikemizu S (2002). A functional and structural basis for TCR cross-reactivity in multiple sclerosis. *Nature Immunology*.

[3] Poole BD, Scofield RH, Harley JB, James JA (2006). Epstein-Barr virus and molecular mimicry in systemic lupus erythematosus. *Autoimmunity*.

[4] Lünemann JD, Jelčić I, Roberts S (2008). EBNA1-specific T cells from patients with multiple sclerosis cross react with myelin antigens and co-produce IFN-*γ* and IL-2. *Journal of Experimental Medicine*.

[5] Pender MP (2003). Infection of autoreactive B lymphocytes with EBV, causing chronic autoimmune diseases. *Trends in Immunology*.

[6] Nemerow GR, Mold C, Schwend VK, Tollefson V, Cooper NR (1987). Identification of gp350 as the viral glycoprotein mediating attachment of Epstein-Barr virus (EBV) to the EBV/C3d receptor of B cells: sequence homology of gp350 and C3 complement fragment C3d. *Journal of Virology*.

[7] Thorley-Lawson DA, Gross A (2004). Persistence of the Epstein-Barr virus and the origins of associated lymphomas. *The New England Journal of Medicine*.

[8] Laichalk LL, Thorley-Lawson DA (2005). Terminal differentiation into plasma cells initiates the replicative cycle of Epstein-Barr virus in vivo. *Journal of Virology*.

[9] Hadinoto V, Shapiro M, Sun CC, Thorley-Lawson DA (2009). The dynamics of EBV shedding implicate a central role for epithelial cells in amplifying viral output. *PLoS Pathogens*.

[10] Souza TA, Stollar BD, Sullivan JL, Luzuriaga K, Thorley-Lawson DA (2005). Peripheral B cells latently infected with Epstein-Barr virus display molecular hallmarks of classical antigen-selected memory B cells. *Proceedings of the National Academy of Sciences of the United States of America*.

[11] Mancao C, Hammerschmidt W (2007). Epstein-Barr virus latent membrane protein 2A is a B-cell receptor mimic and essential for B-cell survival. *Blood*.

[12] Rastelli J, Hömig-Hölzel C, Seagal J (2008). LMP1 signaling can replace CD40 signaling in B cells in vivo and has unique features of inducing class-switch recombination to IgG1. *Blood*.

[13] Roughan JE, Thorley-Lawson DA (2009). The intersection of Epstein-Barr virus with the germinal center. *Journal of Virology*.

[14] Hislop AD, Taylor GS, Sauce D, Rickinson AB (2007). Cellular responses to viral infection in humans: lessons from Epstein-Barr virus. *Annual Review of Immunology*.

[15] Rickinson AB, Kieff E, Knipe DM, Howley PM (2001). Epstein-Barr virus. *Fields Virology. Volume 2*.

[16] Reinherz EL, Weiner HL, Hauser SL (1980). Loss of suppressor T cells in active multiple sclerosis. Analysis with monoclonal antibodies. *The New England Journal of Medicine*.

[17] Bach MA, Tournier E, Phan-Dinh-Tuy F (1980). Deficit of suppressor T cells in active multiple sclerosis. *The Lancet*.

[18] Thompson AJ, Brazil J, Whelan CA, Martin EA, Hutchinson M, Feighery C (1986). Peripheral blood T lymphocyte changes in multiple sclerosis: a marker of disease progression rather than of relapse?. *Journal of Neurology Neurosurgery and Psychiatry*.

[19] Kreuzfelder E, Shen G, Bittorf M (1992). Enumeration of T, B and natural killer peripheral blood cells of patients with multiple sclerosis and controls. *European Neurology*.

[20] Michałowska-Wender G, Wender M (2006). Mononuclear subsets in the peripheral blood of multiple sclerosis patients in relation to results of brain gadolinium—enhancing imaging. *Folia Neuropathologica*.

[21] Pender MP, Csurhes PA, Pfluger CMM, Burrows SR (2011). Decreased CD8+T cell response to Epstein-Barr virus infected B cells in multiple sclerosis is not due to decreased HLA class I expression on B cells or monocytes. *BMC Neurology*.

[22] Veys EM, Hermanns P, Goldstein G, Kung P, Schindler J, Van Wauwe J (1981). Determination of T lymphocyte subpopulations by monoclonal antibodies in rheumatoid arthritis. Influence of immunomodulating agents. *International Journal of Immunopharmacology*.

[23] Fox RI, Fong S, Sabharwal N, Carstens SA, Kung PC, Vaughan JH (1982). Synovial fluid lymphocytes differ from peripheral blood lymphocytes atients with rheumatoid arthritis. *Journal of Immunology*.

[24] Morimoto C, Reinherz EL, Schlossman SF, Schur PH, Mills JA, Steinberg AD (1980). Alterations in immunoregulatory T cell subsets in active systemic lupus erythematosus. *Journal of Clinical Investigation*.

[25] Morimoto C, Reinherz EL, Nadler LM, Distaso JA, Steinberg AD, Schlossman SF (1982). Comparison in T- and B-cell markers in patients with Sjögren's syndrome and systemic lupus erythematosus. *Clinical Immunology and Immunopathology*.

[26] Fox RI, Carstens SA, Fong S, Robinson CA, Howell F, Vaughan JH (1982). Use of monoclonal antibodies to analyze peripheral blood and salivary gland lymphocyte subsets in Sjögren's syndrome. *Arthritis and Rheumatism*.

[27] Whiteside TL, Kumagai Y, Roumm AD, Almendinger R, Rodnan GP (1983). Suppressor cell function and T lymphocyte subpopulations in peripheral blood of patients with progressive systemic sclerosis. *Arthritis and Rheumatism*.

[28] Gustafsson R, Tötterman TH, Klareskog L, Hällgren R (1990). Increase in activated T cells and reduction in suppressor inducer T cells in systemic sclerosis. *Annals of the Rheumatic Diseases*.

[29] O’Gorman MRG, Corrochano V, Roleck J, Donovan M, Pachman LM (1995). Flow cytometric analyses of the lymphocyte subsets in peripheral blood of children with untreated active juvenile dermatomyositis. *Clinical and Diagnostic Laboratory Immunology*.

[30] Aleksza M, Szegedi A, Antal-Szalmás P (2005). Altered cytokine expression of peripheral blood lymphocytes in polymyositis and dermatomyositis. *Annals of the Rheumatic Diseases*.

[31] Moreno-Otero R, Civeira MP, Suou T, Kanof ME, James SP, Jones EA (1994). Reduced numbers of CD8+ T cells and B cell expression of Leu-8 antigen in peripheral blood of patients with primary biliary cirrhosis. *Hepato-Gastroenterology*.

[32] Lindor KD, Wiesner RH, Katzmann JA (1987). Lymphocyte subsets in primary sclerosing cholangitis. *Digestive Diseases and Sciences*.

[33] Godin NJ, Sachar DB, Winchester R (1984). Loss of suppressor T-cells in active inflammatory bowel disease. *Gut*.

[34] Learmonth RP, Pihl E, Johnson WR, Barnett MA, McDermott FT, Hughes ES (1984). Altered blood lymphocyte subclasses in patients with ulcerative colitis. *Australian and New Zealand Journal of Surgery*.

[35] Kokelj F, Perticarari S, Presani G, Trevisan G (1983). An analysis of T-lymphocyte subpopulations in psoriasis using monoclonal antibodies. *Acta Dermato-Venereologica*.

[36] D’Amelio R, Frati C, Fattorossi A, Aiuti F (1990). Peripheral T-cell subset imbalance in patients with vitiligo and in their apparently healthy first-degree relatives. *Annals of Allergy*.

[37] Pichler R, Sfetsos K, Badics B, Gutenbrunner S, Berg J, Auböck J (2009). Lymphocyte imbalance in vitiligo patients indicated by elevated CD4+/CD8+ T-cell ratio. *Wiener Medizinische Wochenschrift*.

[38] Hovmark A, Asbrink E (1982). An immunological study of patients with bullous pemphigiod. *Acta Dermato-Venereologica*.

[39] Guillot B, Laure H, Andary M (1990). Immunological studies in bullous pemphigoid: cellular immunity before and after treatment with corticosteroid and plasma exchanges. *Transfusion Science*.

[40] Luckac J, Burek B, Kusić Z (1993). Peripheral blood lymphocyte populations and phagocytic functions in patients with active alopecia areata. *Acta Medica Croatica*.

[41] Sanderson JE, Koech D, Iha D, Ojiambo HP (1985). T-lymphocyte subsets in idiopathic dilated cardiomyopathy. *American Journal of Cardiology*.

[42] Bozkurt A, Canataroğlu A, Cetiner S, Dönmez Y, Usal A, Demirtaş M (2001). Lymphocyte subsets in patients with idiopathic dilated cardiomyopathy. *Anadolu Kardiyoloji Dergisi*.

[43] Buschard K, Ropke C, Madsbad S, Mehlsen J, Sørensen TB, Rygaard J (1983). Alterations of peripheral T-lymphocyte subpopulations in patients with insulin-dependent (type 1) diabetes mellitus. *Journal of Clinical and Laboratory Immunology*.

[44] Galluzzo A, Giordano C, Rubino G, Bompiani GD (1984). Immunoregulatory T-lymphocyte subset deficiency in newly diagnosed type 1 (insulin-dependent) diabetes mellitus. *Diabetologia*.

[45] Johnston C, Alviggi L, Millward BA, Leslie RDG, Pyke DA, Vergani D (1988). Alterations in T-lymphocyte subpopulations in type I diabetes. Exploration of genetic influence in identical twins. *Diabetes*.

[46] Kaaba SA, Al-Harbi SA (1995). Abnormal lymphocyte subsets in Kuwaiti patients with type-1 insulin-dependent diabetes mellitus and their first-degree relatives. *Immunology Letters*.

[47] Thielemans C, Vanhaelst L, De Waele M (1981). Autoimmune thyroiditis: a condition related to a decrease in T-suppressor cells. *Clinical Endocrinology*.

[48] Xia N, Zhou S, Liang Y (2006). CD4^+^ T cells and the Th1/Th2 imbalance are implicated in the pathogenesis of Graves’ ophthalmopathy. *International Journal of Molecular Medicine*.

[49] Iwatani Y, Amino N, Hidaka Y (1992). Decreases in *αβ* T cell receptor negative T cells and CD8 cells, and an increase in CD4^+^ CD8^+^ cells in active Hashimoto’s disease and subacute thyroiditis. *Clinical and Experimental Immunology*.

[50] Berrih S, Gaud C, Bach MA, Le Brigand H, Binet JP, Bach JF (1981). Evaluation of T cell subsets in myasthenia gravis using anti-T cell monoclonal antibodies. *Clinical and Experimental Immunology*.

[51] Skolnik PR, Lisak RP, Zweiman B (1982). Monoclonal antibody analysis of blood T-cell subsets in myasthenia gravis. *Annals of Neurology*.

[52] Chatenoud L, Bach MA (1981). Abnormalities of T-cell subsets in glomerulonephritis and systemic lupus erythematosus. *Kidney International*.

[53] Fornasieri A, Sinico R, Fiorini G (1983). T-lymphocyte subsets in primary and secondary glomerulonephritis. *Proceedings of the European Dialysis and Transplant Association*.

[54] Imamura N, Fujimura K, Kuramoto A (1984). Lymphocyte subpopulations in pernicious anemia. *The New England Journal of Medicine*.

[55] Gogos CA, Kapatais-Zoumbos KN, Zoumbos NC (1986). Lymphocyte subpopulations in megaloblastic anaemia due to vitamin B-12 deficiency. *Scandinavian Journal of Haematology*.

[56] Iwatani Y, Amino N, Mori H (1983). T lymphocyte subsets in autoimmune thyroid diseases and subacute thyroiditis detected with monoclonal antibodies. *Journal of Clinical Endocrinology and Metabolism*.

[57] Bonnyns M, Bentin J, Devetter G, Duchateau J (1983). Heterogeneity of immunoregulatory T cells in human thyroid autoimmunity: influence of thyroid status. *Clinical and Experimental Immunology*.

[58] Booss J, Esiri MM, Tourtellotte WW, Mason DY (1983). Immunohistological analysis of T lymphocyte subsets in the central nervous system in chronic progressive multiple sclerosis. *Journal of the Neurological Sciences*.

[59] Hoshino Y, Morishima T, Kimura H, Nishikawa K, Tsurumi T, Kuzushima K (1999). Antigen-driven expansion and contraction of CD8+-activated T cells in primary EBV infection. *Journal of Immunology*.

[60] Yajima M, Imadome KI, Nakagawa A (2008). A new humanized mouse model of Epstein-Barr virus infection that reproduces persistent infection, lymphoproliferative disorder, and cell-mediated and humoral immune responses. *Journal of Infectious Diseases*.

[61] Strowig T, Gurer C, Ploss A (2009). Priming of protective T cell responses against virus-induced tumors in mice with human immune system components. *Journal of Experimental Medicine*.

[62] Amadori A, Zamarchi R, De Silvestro G (1995). Genetic control of the CD4/CD8 T-cell ratio in humans. *Nature Medicine*.

[63] Ferreira MAR, Mangino M, Brumme CJ (2010). Quantitative trait loci for CD4:CD8 lymphocyte ratio are associated with risk of type 1 diabetes and HIV-1 immune control. *American Journal of Human Genetics*.

[64] Jabs DA, Arnett FC, Bias WB, Beale MG (1986). Familial abnormalities of lymphocyte function in a large Sjögren’s syndrome kindred. *Journal of Rheumatology*.

[65] Johansen M, Elling P, Elling H, Olsson A (1995). A genetic approach to the aetiology of giant cell arteritis: depletion of the CD8+ T-lymphocyte subset in relatives of patients with polymyalgia rheumatica and arteritis temporalis. *Clinical and Experimental Rheumatology*.

[66] Tugume SB, Piwowar EM, Lutalo T (1995). Hematological reference ranges among healthy Ugandans. *Clinical and Diagnostic Laboratory Immunology*.

[67] Menard D, Mandeng MJ, Tothy MB, Kelembho EK, Gresenguet G, Talarmin A (2003). Immunohematological reference ranges for adults from the Central African Republic. *Clinical and Diagnostic Laboratory Immunology*.

[68] Uppal SS, Verma S, Dhot PS (2003). Normal values of CD4 and CD8 lymphocyte subsets in healthy Indian adults and the effects of sex, age, ethnicity, and smoking. *Cytometry Part B*.

[69] Jiang W, Kang L, Lu HZ (2004). Normal values for CD4 and CD8 lymphocyte subsets in healthy Chinese adults from Shanghai. *Clinical and Diagnostic Laboratory Immunology*.

[70] Jentsch-Ullrich K, Koenigsmann M, Mohren M, Franke A (2005). Lymphocyte subsets’ reference ranges in an age- and gender-balanced population of 100 healthy adults—a monocentric German study. *Clinical Immunology*.

[71] Ho PC, Tang GWK, Lawton JWM (1991). Lymphocyte subsets in patients with oestrogen deficiency. *Journal of Reproductive Immunology*.

[72] Comans-Bitter WM, de Groot R, van den Beemd R (1997). Immunophenotyping of blood lymphocytes in childhood: reference values for lymphocyte subpopulations. *Journal of Pediatrics*.

[73] Hall MA, Ahmadi KR, Norman P (2000). Genetic influence on peripheral blood T lymphocyte levels. *Genes and Immunity*.

[74] Pender MP, Csurhes PA, Pfluger CMM, Burrows SR CD8 T cell deficiency impairs control of Epstein-Barr virus and worsens with age in multiple sclerosis.

[75] Hersey P, Bradley M, Hasic E, Haran G, Edwards A, McCarthy WH (1983). Immunological effects of solarium exposure. *The Lancet*.

[76] Hersey P, Haran G, Hasic E, Edwards A (1983). Alteration of T cell subsets and induction of suppressor T cell activity in normal subjects after exposure to sunlight. *Journal of Immunology*.

[77] Falkenbach A, Sedlmeyer A (1997). Travel to sunny countries is associated with changes in immunological parameters. *Photodermatology Photoimmunology and Photomedicine*.

[78] Veldman CM, Cantorna MT, DeLuca HF (2000). Expression of 1,25-dihydroxyvitamin D_3_ receptor in the immune system. *Archives of Biochemistry and Biophysics*.

[79] Nonnecke BJ, Franklin ST, Reinhardt TA, Horst RL (1993). In vitro modulation of proliferation and phenotype of resting and mitogen-stimulated bovine mononuclear leukocytes by 1,25-dihydroxyvitamin D_3_. *Veterinary Immunology and Immunopathology*.

[80] Žofková I, Kancheva RL (1997). The effect of 1,25(OH)_2_ vitamin D_3_ on CD4+/CD8+ subsets of T lymphocytes in postmenopausal women. *Life Sciences*.

[81] Çakmak FN, Erol M, Ergül P, Yalçýner A (1999). T lymphocytes and vitamins. *Journal of Pediatrics*.

[82] Ulett G (1948). Geographic distribution of multiple sclerosis. *Diseases of the Nervous System*.

[83] Staples JA, Ponsonby AL, Lim LLY, McMichael AJ (2003). Ecologic analysis of some immune-related disorders, including type 1 diabetes, in Australia: latitude, regional ultraviolet radiation, and disease prevalence. *Environmental Health Perspectives*.

[84] Vieira VM, Hart JE, Webster TF (2010). Association between residences in U.S. northern latitudes and rheumatoid arthritis: a spatial analysis of the nurses’ health study. *Environmental Health Perspectives*.

[85] Munger KL, Levin LI, Hollis BW, Howard NS, Ascherio A (2006). Serum 25-hydroxyvitamin D levels and risk of multiple sclerosis. *Journal of the American Medical Association*.

[86] Arnson Y, Amital H, Agmon-Levin N (2011). Serum 25-OH vitamin D concentrations are linked with various clinical aspects in patients with systemic sclerosis: a retrospective cohort study and review of the literature. *Autoimmunity Reviews*.

[87] Peelen E, Knippenberg S, Muris A-H (2011). Effects of vitamin D on the peripheral adaptive immune system: a review. *Autoimmunity Reviews*.

[88] Haahr S, Höllsberg P (2006). Multiple sclerosis is linked to Epstein-Barr virus infection. *Reviews in Medical Virology*.

[89] Bray PF, Bloomer LC, Salmon VC (1983). Epstein-Barr virus infection and antibody synthesis in patients with multiple sclerosis. *Archives of Neurology*.

[90] Wandinger KP, Jabs W, Siekhaus A (2000). Association between clinical disease activity and Epstein-Barr virus reactivation in MS. *Neurology*.

[91] Ascherio A, Munger KL (2007). Environmental risk factors for multiple sclerosis. Part I: the role of infection. *Annals of Neurology*.

[92] Levin LI, Munger KL, O’Reilly EJ, Falk KI, Ascherio A (2010). Primary infection with the Epstein-Barr virus and risk of multiple sclerosis. *Annals of Neurology*.

[93] Pender MP (2011). The essential role of Epstein-Barr virus in the pathogenesis of multiple sclerosis. *Neuroscientist*.

[94] Pohl D, Krone B, Rostasy K (2006). High seroprevalence of Epstein-Barr virus in children with multiple sclerosis. *Neurology*.

[95] Banwell B, Krupp L, Kennedy J (2007). Clinical features and viral serologies in children with multiple sclerosis: a multinational observational study. *The Lancet Neurology*.

[96] Lünemann JD, Huppke P, Roberts S, Brück W, Gärtner J, Münz C (2008). Broadened and elevated humoral immune response to EBNA1 in pediatric multiple sclerosis. *Neurology*.

[97] James JA, Kaufman KM, Farris AD, Taylor-Albert E, Lehman TJA, Harley JB (1997). An increased prevalence of Epstein-Barr virus infection in young patients suggests a possible etiology for systemic lupus erythematosus. *Journal of Clinical Investigation*.

[98] James JA, Neas BR, Moser KL (2001). Systemic lupus erythematosus in adults is associated with previous Epstein-Barr virus exposure. *Arthritis and Rheumatism*.

[99] Thomas D, Karachaliou F, Kallergi K (2008). Herpes virus antibodies seroprevalence in children with autoimmune thyroid disease. *Endocrine*.

[100] Nagata K, Fukata S, Kanai K (2011). The influence of epstein-barr virus reactivation in patients with Graves' disease. *Viral Immunology*.

[101] Harada S, Greally J, Davis J (1985). Epstein-Barr virus specific antibodies in patients with coeliac disease. *Irish Journal of Medical Science*.

[102] Hochberg D, Souza T, Catalina M, Sullivan JL, Luzuriaga K, Thorley-Lawson DA (2004). Acute infection with Epstein-Barr virus targets and overwhelms the peripheral memory B-cell compartment with resting, latently infected cells. *Journal of Virology*.

[103] Thacker EL, Mirzaei F, Ascherio A (2006). Infectious mononucleosis and risk for multiple sclerosis: a meta-analysis. *Annals of Neurology*.

[104] Haahr S, Plesner AM, Vestergaard BF, Höllsberg P (2004). A role of late Epstein-Barr virus infection in multiple sclerosis. *Acta Neurologica Scandinavica*.

[105] Crowcroft NS, Vyse A, Brown DWG, Strachan DP (1998). Epidemiology of Epstein-Barr virus infection in pre-adolescent children: application of a new salivary method in Edinburgh, Scotland. *Journal of Epidemiology and Community Health*.

[106] Kondrashova A, Viskari H, Haapala A-M (2008). Serological evidence of thyroid autoimmunity among schoolchildren in two different socioeconomic environments. *Journal of Clinical Endocrinology and Metabolism*.

[107] Tosato G, Steinberg AD, Blaese RM (1981). Defective EBV-specific suppressor T-cell function in rheumatoid arthritis. *The New England Journal of Medicine*.

[108] Gaston JSH, Rickinson AB, Epstein MA (1982). Epstein-Barr virus-specific cytotoxic T cell responses in rheumatoid arthritis patients. *Rheumatology International*.

[109] Tosato G, Steinberg AD, Yarchoan R (1984). Abnormally elevated frequency of Epstein-Barr virus-infected B cells in the blood of patients with rheumatoid arthritis. *Journal of Clinical Investigation*.

[110] Tsokos GC, Magrath IT, Balow JE (1983). Epstein-Barr virus induces normal B cell responses but defective suppressor T cell responses in patients with systemic lupus erythematosus. *Journal of Immunology*.

[111] Whittingham S, McNeilage J, Mackay IR (1985). Primary Sjögren’s syndrome after infectious mononucleosis. *Annals of Internal Medicine*.

[112] Yamaoka K, Miyasaka N, Yamamoto K (1988). Possible involvement of Epstein-Barr virus in polyclonal B cell activation in Sjögren’s syndrome. *Arthritis and Rheumatism*.

[113] Craig JC, Hawkins SA, Swallow MW (1985). Subsets of T lymphocytes in relation to T lymphocyte function in multiple sclerosis. *Clinical and Experimental Immunology*.

[114] Pender MP, Csurhes PA, Lenarczyk A, Pfluger CMM, Burrows SR (2009). Decreased T cell reactivity to Epstein-Barr virus infected lymphoblastoid cell lines in multiple sclerosis. *Journal of Neurology, Neurosurgery and Psychiatry*.

[115] James SP, Jones EA, Hoofnagle JH, Strober W (1985). Circulating activated B cells in primary biliary cirrhosis. *Journal of Clinical Immunology*.

[116] Shore A, Klock R, Lee P, Snow KM, Keystone EC (1989). Impaired late suppression of Epstein-Barr virus (EBV)-induced immunoglobulin synthesis: a common feature of autoimmune disease. *Journal of Clinical Immunology*.

[117] Yi JS, Cox MA, Zajac AJ (2010). T-cell exhaustion: characteristics, causes and conversion. *Immunology*.

[118] Saito I, Servenius B, Compton T, Fox RI (1989). Detection of Epstein-Barr virus DNA by polymerase chain reaction in blood and tissue biopsies from patients with Sjögren’s syndrome. *Journal of Experimental Medicine*.

[119] Pflugfelder SC, Crouse C, Pereira I, Atherton S (1990). Amplification of Epstein-Barr virus genomic sequences in blood cells, lacrimal glands, and tears from primary Sjögren’s syndrome patients. *Ophthalmology*.

[120] Balandraud N, Meynard JB, Auger I (2003). Epstein-Barr virus load in the peripheral blood of patients with rheumatoid arthritis: accurate quantification using real-time polymerase chain reaction. *Arthritis and Rheumatism*.

[121] Kang I, Quan T, Nolasco H (2004). Defective control of latent Epstein-Barr virus infection in systemic lupus erythematosus. *Journal of Immunology*.

[122] Morshed SA, Nishioka M, Saito I, Komiyama K, Moro I (1992). Increased expression of Epstein-Barr virus in primary biliary cirrhosis patients. *Gastroenterologia Japonica*.

[123] Sankaran-Walters S, Ransibrahmanakul K, Grishina I (2011). Epstein-Barr virus replication linked to B cell proliferation in inflamed areas of colonic mucosa of patients with inflammatory bowel disease. *Journal of Clinical Virology*.

[124] Gross AJ, Hochberg D, Rand WM, Thorley-Lawson DA (2005). EBV and systemic lupus erythematosus: a new perspective. *Journal of Immunology*.

[125] Catalano MA, Carson DA, Slovin SF, Richman DD, Vaughan JH (1979). Antibodies to Epstein-Barr virus-determined antigens in normal subjects and in patients with seropositive rheumatoid arthritis. *Proceedings of the National Academy of Sciences of the United States of America*.

[126] Sumaya CV, Myers LW, Ellison GW (1980). Epstein-Barr virus antibodies in multiple sclerosis. *Archives of Neurology*.

[127] Vergnon JM, de Thé G, Weynants P, Vincent M, Mornex JF, Brune J (1984). Cryptogenic fibrosing alveolitis and Epstein-Barr virus: an association?. *The Lancet*.

[128] Origgi L, Hu C, Bertetti E (1988). Antibodies to Epstein-Barr virus and cytomegalovirus in primary Sjögren's syndrome. *Bollettino dell'Istituto Sieroterapico Milanese*.

[129] Andre PM, Le Pogamp P, Griffais R, Chevet D, Ramee MP (1990). Is Epstein-Barr virus involved in primary IgA nephropathy?. *Nephron*.

[130] Vrbikova J, Janatkova I, Zamrazil V, Tomiska R, Fucikova T (1996). Epstein-Barr virus serology in patients with autoimmune thyroiditis. *Experimental and Clinical Endocrinology and Diabetes*.

[131] Ascherio A, Munger KL, Lennette ET (2001). Epstein-Barr virus antibodies and risk of multiple sclerosis: a prospective study. *Journal of the American Medical Association*.

[132] Sundström P, Juto P, Wadell G (2004). An altered immune response to Epstein-Barr virus in multiple sclerosis: a prospective study. *Neurology*.

[133] Levin LI, Munger KL, Rubertone MV (2005). Temporal relationship between elevation of Epstein-Barr virus antibody titers and initial onset of neurological symptoms in multiple sclerosis. *Journal of the American Medical Association*.

[134] Lünemann JD, Edwards N, Muraro PA (2006). Increased frequency and broadened specificity of latent EBV nuclear antigen-1-specific T cells in multiple sclerosis. *Brain*.

[135] Babcock GJ, Decker LL, Freeman RB, Thorley-Lawson DA (1999). Epstein-Barr virus-infected resting memory B cells, not proliferating lymphoblasts, accumulate in the peripheral blood of immunosuppressed patients. *Journal of Experimental Medicine*.

[136] Gulley ML, Tang W (2008). Laboratory assays for Epstein-Barr virus-related disease. *Journal of Molecular Diagnostics*.

[137] Spieker T, Herbst H (2000). Distribution and phenotype of Epstein-Barr virus-infected cells in inflammatory bowel disease. *American Journal of Pathology*.

[138] Pflugfelder SC, Crouse CA, Monroy D, Yen M, Rowe M, Atherton SS (1993). Epstein-Barr virus and the lacrimal gland pathology of Sjögren’s syndrome. *American Journal of Pathology*.

[139] Wen S, Shimizu N, Yoshiyama H, Mizugaki Y, Shinozaki F, Takada K (1996). Association of Epstein-Barr virus (EBV) with Sjögren’s syndrome: differential EBV expression between epithelial cells and lymphocytes in salivary glands. *American Journal of Pathology*.

[140] Yanai H, Shimizu N, Nagasaki S, Mitani N, Okita K (1999). Epstein-Barr virus infection of the colon with inflammatory bowel disease. *American Journal of Gastroenterology*.

[141] Serafini B, Rosicarelli B, Franciotta D (2007). Dysregulated Epstein-Barr virus infection in the multiple sclerosis brain. *Journal of Experimental Medicine*.

[142] Cavalcante P, Serafini B, Rosicarelli B (2010). Epstein-Barr virus persistence and reactivation in myasthenia gravis thymus. *Annals of Neurology*.

[143] Iwama H, Horikoshi S, Shirato I, Tomino Y (1998). Epstein-Barr virus detection in kidney biopsy specimens correlates with glomerular mesangial injury. *American Journal of Kidney Diseases*.

[144] Stewart JP, Egan JJ, Ross AJ (1999). The detection of Epstein-Barr virus DNA in lung tissue from patients with idiopathic pulmonary fibrosis. *American Journal of Respiratory and Critical Care Medicine*.

[145] Hoffman RW (2004). T cells in the pathogenesis of systemic lupus erythematosus. *Clinical Immunology*.

[146] Matsubayashi S, Tamai H, Morita T (1990). Hashimoto’s thyroiditis manifesting monoclonal lymphocytic infiltration. *Clinical and Experimental Immunology*.

[147] Pablos JL, Carreira PE, Morillas L, Montalvo G, Ballestin C, Gomez-Reino JJ (1994). Clonally expanded lymphocytes in the minor salivary glands of Sjögren’s syndrome patients without lymphoproliferative disease. *Arthritis and Rheumatism*.

[148] Stott DI, Hiepe F, Hummel M, Steinhauser G, Berek C (1998). Antigen-driven clonal proliferation of B cells within the target tissue of an autoimmune disease. The salivary glands of patients with Sjögren’s syndrome. *Journal of Clinical Investigation*.

[149] Gause A, Gundlach K, Zdichavsky M (1995). The B lymphocyte in rheumatoid arthritis: analysis of rearranged V*κ* genes from B cells infiltrating the synovial membrane. *European Journal of Immunology*.

[150] Schröder AE, Greiner A, Seyfert C, Berek C (1996). Differentiation of B cells in the nonlymphoid tissue of the synovial membrane of patients with rheumatoid arthritis. *Proceedings of the National Academy of Sciences of the United States of America*.

[151] Qin Y, Duquette P, Zhang Y, Talbot P, Poole R, Antel J (1998). Clonal expansion and somatic hypermutation of V_H_ genes of B cells from cerebrospinal fluid in multiple sclerosis. *Journal of Clinical Investigation*.

[152] Sugimura T, Shiokawa S, Haraoka S (2003). Local antigen-driven oligoclonal expansion of B cells in the liver portal areas of patients with primary biliary cirrhosis. *Liver International*.

[153] Bradshaw EM, Orihuela A, McArdel SL (2007). A local antigen-driven humoral response is present in the inflammatory myopathies. *Journal of Immunology*.

[154] Sfikakis PP, Karali V, Lilakos K, Georgiou G, Panayiotidis P (2009). Clonal expansion of B-cells in human systemic lupus erythematosus: evidence from studies before and after therapeutic B-cell depletion. *Clinical Immunology*.

[155] McLachlan SM, Dickinson AM, Malcolm A (1983). Thyroid autoantibody synthesis by cultures of thyroid and peripheral blood lymphocytes. I. Lymphocyte markers and response to pokeweed mitogen. *Clinical and Experimental Immunology*.

[156] Armengol MP, Juan M, Lucas-Martín A (2001). Thyroid autoimmune disease: demonstration of thyroid antigen-specific B cells and recombination-activating gene expression in chemokine-containing active intrathyroidal germinal centers. *American Journal of Pathology*.

[157] Salomonsson S, Jonsson MV, Skarstein K (2003). Cellular basis of ectopic germinal center formation and autoantibody production in the target organ of patients with Sjögren’s syndrome. *Arthritis and Rheumatism*.

[158] Björkland A, Lööf L, Mendel-Hartvig I, Tötterman TH (1994). Primary biliary cirrhosis: high proportions of B cells in blood and liver tissue produce anti-mitochondrial antibodies of several Ig classes. *Journal of Immunology*.

[159] Wardemann H, Yurasov S, Schaefer A, Young JW, Meffre E, Nussenzweig MC (2003). Predominant autoantibody production by early human B cell precursors. *Science*.

[160] Garzelli C, Taub FE, Scharff JE, Prabhakar BS, Ginsberg-Fellner F, Notkins AL (1984). Epstein-Barr virus-transformed lymphocytes produce monoclonal autoantibodies that react with antigens in multiple organs. *Journal of Virology*.

[161] Schwartz-Albiez R, Monteiro RC, Rodriguez M, Binder CJ, Shoenfeld Y (2009). Natural antibodies, intravenous immunoglobulin and their role in autoimmunity, cancer and inflammation. *Clinical and Experimental Immunology*.

[162] Londei M, Bottazzo GF, Feldmann M (1985). Human T-cell clones from autoimmune thyroid glands: specific recognition of autologous thyroid cells. *Science*.

[163] Dayan CM, Londei M, Corcoran AE (1991). Autoantigen recognition by thyroid-infiltrating T cells in Graves disease. *Proceedings of the National Academy of Sciences of the United States of America*.

[164] Shimoda S, van de Water J, Ansari A (1998). Identification and precursor frequency analysis of a common T cell epitope motif in mitochondrial autoantigens in primary biliary cirrhosis. *Journal of Clinical Investigation*.

[165] Kita H, Matsumura S, He XS (2002). Quantitative and functional analysis of PDC-E2-specific autoreactive cytotoxic T lymphocytes in primary biliary cirrhosis. *Journal of Clinical Investigation*.

[166] Namekawa T, Kuroda K, Kato T (1995). Identification of Ro(SSA) 52 kDa reactive T cells in labial salivary glands from patients with Sjögren’s syndrome. *Journal of Rheumatology*.

[167] Molberg Ø, Mcadam SN, Körner R (1998). Tissue transglutaminase selectively modifies gliadin peptides that are recognized by gut-derived T cells in celiac disease. *Nature Medicine*.

[168] Tabi Z, McCombe PA, Pender MP (1994). Apoptotic elimination of V*β*8.2^+^ cells from the central nervous system during recovery from experimental autoimmune encephalomyelitis induced by the passive transfer of V*β*8.2^+^ encephalitogenic T cells. *European Journal of Immunology*.

[169] Pender MP (1998). Genetically determined failure of activation-induced apoptosis of autoreactive T cells as a cause of multiple sclerosis. *The Lancet*.

[170] Hadfield G (1939). The primary histological lesion of regional ileitis. *The Lancet*.

[171] Carlsen HS, Baekkevold ES, Johansen FE, Haraldsen G, Brandtzaeg P (2002). B cell attracting chemokine 1 (CXCL13) and its receptor CXCR5 are expressed in normal and aberrant gut associated lymphoid tissue. *Gut*.

[172] Hadziyannis S, Scheuer PJ, Feizi T, Naccarato R, Doniach D, Sherlock S (1970). Immunological and histological studies in primary biliary cirrhosis. *Journal of Clinical Pathology*.

[173] Soderstrom N, Biorklund A (1974). Organization of the invading lymphoid tissue in human lymphoid thyroiditis. *Scandinavian Journal of Immunology*.

[174] Young CL, Adamson TC, Vaughan JH, Fox RI (1984). Immunohistologic characterization of synovial membrane lymphocytes in rheumatoid arthritis. *Arthritis and Rheumatism*.

[175] Randen I, Mellbye OJ, Forre O, Natvig JB (1995). The identification of germinal centres and follicular dendritic cell networks in rheumatoid synovial tissue. *Scandinavian Journal of Immunology*.

[176] Cañete JD, Santiago B, Cantaert T (2007). Ectopic lymphoid neogenesis in psoriatic arthritis. *Annals of the Rheumatic Diseases*.

[177] Campbell DA, Poulter LW, Janossy G, Du Bois RM (1985). Immunohistological analysis of lung tissue from patients with cryptogenic fibrosing alveolitis suggesting local expression of immune hypersensitivity. *Thorax*.

[178] Wallace WAH, Howie SEM, Krajewski AS, Lamb D (1996). The immunological architecture of B-lymphocyte aggregates in cryptogenic fibrosing alveolitis. *Journal of Pathology*.

[179] Amft N, Curnow SJ, Scheel-Toellner D (2001). Ectopic expression of the B cell-attracting chemokine BCA-1 (CXCL13) on endothelial cells and within lymphoid follicles contributes to the establishment of germinal center-like structures in Sjögren’s syndrome. *Arthritis and Rheumatism*.

[180] Serafini B, Rosicarelli B, Magliozzi R, Stigliano E, Aloisi F (2004). Detection of ectopic B-cell follicles with germinal centers in the meninges of patients with secondary progressive multiple sclerosis. *Brain Pathology*.

[181] Magliozzi R, Howell O, Vora A (2007). Meningeal B-cell follicles in secondary progressive multiple sclerosis associate with early onset of disease and severe cortical pathology. *Brain*.

[182] Cohen CD, Calvaresi N, Armelloni S (2005). CD20-positive infiltrates in human membranous glomerulonephritis. *Journal of Nephrology*.

[183] Heller F, Lindenmeyer MT, Cohen CD (2007). The contribution of B cells to renal interstitial inflammation. *American Journal of Pathology*.

[184] De Padilla CML, Vallejo AN, Lacomis D, Mcnallan K, Reed AM (2009). Extranodal lymphoid microstructures in inflamed muscle and disease severity of new-onset juvenile dermatomyositis. *Arthritis and Rheumatism*.

[185] Levine GD, Rosai J (1978). Thymic hyperplasia and neoplasia: a review of current concepts. *Human Pathology*.

[186] Thomas JA, Willcox HNA, Newsom-Davis J (1982). Immunohistological studies of the thymus in myasthenia gravis. Correlation with clinical state and thymocyte culture responses. *Journal of Neuroimmunology*.

[187] Roxanis I, Micklem K, McConville J, Newsom-Davis J, Willcox N (2002). Thymic myoid cells and germinal center formation in myasthenia gravis; possible roles in pathogenesis. *Journal of Neuroimmunology*.

[188] Kojima M, Nakamura S, Morishita Y (2000). Reactive follicular hyperplasia in the lymph node lesions from systemic lupus erythematosus patients: a clinicopathological and immunohistological study of 21 cases. *Pathology International*.

[189] Sadovnick AD (2012). Genetic background of multiple sclerosis. *Autoimmunity Reviews*.

[190] Ballestar E (2011). Epigenetic alterations in autoimmune rheumatic diseases. *Nature Reviews Rheumatology*.

[191] Bias WB, Reveille JD, Beaty TH, Meyers DA, Arnett FC (1986). Evidence that autoimmunity in man is a Mendelian dominant trait. *American Journal of Human Genetics*.

[192] McCombe PA, Chalk JB, Pender MP (1990). Familial occurrence of multiple sclerosis with thyroid disease and systemic lupus erythematosus. *Journal of the Neurological Sciences*.

[193] Lin JP, Cash JM, Doyle SZ (1998). Familial clustering of rheumatoid arthritis with other autoimmune diseases. *Human Genetics*.

[194] Henderson RD, Bain CJ, Pender MP (2000). The occurrence of autoimmune diseases in patients with multiple sclerosis and their families. *Journal of Clinical Neuroscience*.

[195] Barcellos LF, Kamdar BB, Ramsay PP (2006). Clustering of autoimmune diseases in families with a high-risk for multiple sclerosis: a descriptive study. *The Lancet Neurology*.

[196] Thorsby E, Lie BA (2005). HLA associated genetic predisposition to autoimmune diseases: genes involved and possible mechanisms. *Transplant Immunology*.

[197] Pender MP (2009). Does Epstein-Barr virus infection in the brain drive the development of multiple sclerosis. *Brain*.

[198] Pender MP (2009). Preventing and curing multiple sclerosis by controlling Epstein-Barr virus infection. *Autoimmunity Reviews*.

[199] Sokal EM, Hoppenbrouwers K, Vandermeulen C (2007). Recombinant gp350 vaccine for infectious mononucleosis: a phase 2, randomized, double-blind, placebo-controlled trial to evaluate the safety, immunogenicity, and efficacy of an Epstein-Barr virus vaccine in healthy young adults. *Journal of Infectious Diseases*.

[200] Haque T, Johannessen I, Dombagoda D (2006). A mouse monoclonal antibody against Epstein-Barr virus envelope glycoprotein 350 prevents infection both in Vitro and in Vivo. *Journal of Infectious Diseases*.

[201] Savoldo B, Goss JA, Hammer MM (2006). Treatment of solid organ transplant recipients with autologous Epstein Barr virus-specific cytotoxic T lymphocytes (CTLs). *Blood*.

[202] Nanjappa SG, Kim EH, Suresh M (2011). Immunotherapeutic effects of IL-7 during a chronic viral infection in mice. *Blood*.

[203] Mei YP, Zhu XF, Zhou JM, Huang H, Deng R, Zeng YX (2006). siRNA targeting LMP1-induced apoptosis in EBV-positive lymphoma cells is associated with inhibition of telomerase activity and expression. *Cancer Letters*.

[204] Li N, Thompson S, Schultz DC (2010). Discovery of selective inhibitors against EBNA1 via high throughput in silico virtual screening. *PLoS One*.

[205] Dreyfus DH (2011). Autoimmune disease: a role for new anti-viral therapies?. *Autoimmunity Reviews*.

[206] Jin B, Ni H, Geshang Q, Li Y, Shen W, Shi H (2011). HLA-DR4 antigen and idiopathic dilated cardiomyopathy susceptibility: a meta-analysis involving 11,761 subjects. *Tissue Antigens*.

[207] Invernizzi P, Selmi C, Poli F (2008). Human leukocyte antigen polymorphisms in Italian primary biliary cirrhosis: a multicenter study of 664 patients and 1992 healthy controls. *Hepatology*.

[208] Ahmad T, Marshall SE, Jewell D (2006). Genetics of inflammatory bowel disease: the role of the HLA complex. *World Journal of Gastroenterology*.

